# Lymphatic Preconditioning for Preservation of Lymphatic Function

**DOI:** 10.3390/ijms27209125

**Published:** 2026-10-15

**Authors:** Shahnur Ahmed, Ganesh Mohan, Angad S. Sidhu, Daniel J. Konig, Christopher A. Subi-Kasozi, Steven Sullivan, Miguel D. Jorge, Kristen N. Calabrese, Helen C. Sinex, Joy Garrett, Joseph R. Dynlacht, Mithun Sinha, Aladdin H. Hassanein

**Affiliations:** 1 Division of Plastic Surgery, Indiana University School of Medicine, Indianapolis, IN 46202, USA; 2 Department of Radiation Medicine, Indiana University School of Medicine, Indianapolis, IN 46202, USA

**Keywords:** murine hindlimb, radiation, lymphedema, lymphadenectomy, negative pressure therapy, prevention

## Abstract

Lymphedema is chronic swelling from lymphatic dysfunction. The vascular delay phenomenon is used in flap reconstruction to increase flap viability through staged selective partial disruption of blood supply to enhance flap survival, followed by flap transfer 7–10 days later. A similar corollary for lymphatics remains unknown. The purpose of this study was to evaluate whether lymphatic preconditioning with staged disruption of lymphatics in the murine hindlimb lymphedema model can be protective against lymphedema. C57BL/6 mice underwent 20 Gy hindlimb irradiation. Group 1 (immediate lymphatic disruption) underwent a circumferential skin incision, complete lymphatic disruption, and popliteal lymph node excision in a single-stage operation. Group 2 (delayed lymphatic disruption) underwent a semi-circumferential inguinal skin incision and dominant lymphatic transection and, 7 days later, received a circumferential skin incision, complete lymphatic transection, and popliteal lymphadenectomy. Paw thickness was measured weekly. Near-infrared laser lymphangiography and immunohistochemistry were performed on D35. Group 2 had a lower average paw thickness (*p* = 0.0008) and greater lymphatic clearance signal intensity at 24 h (*p* = 0.0035). Immunohistochemistry indicated an abundance of the lymphatic endothelial-specific marker podoplanin in Group 2 compared to Group 1 (*p* = 0.03). This study demonstrates evidence for a novel concept of a lymphatic delay phenomenon parallel to the well-known vascular delay phenomenon.

## 1. Introduction

Lymphedema is chronic swelling from lymphatic dysfunction [1,2,3,4]. Injury to lymphatic vessels during lymphadenectomy during cancer management results in an inflammatory cascade leading to irreversible fluid and fibroadipose deposition [1,2,4]. Lymphedema affects up to 10 million individuals in the United States and up to 250 million individuals worldwide [2,5]. The inflammatory cascade with lymphedema results in fibroadipose deposition in addition to fluid and has proven difficult to reverse [2,6,7,8,9,10,11,12,13]. There is no cure for lymphedema [1,2,3,4,5,14,15]. Therefore, there has been a recent focus on clinical and experimental approaches to prevent lymphedema [1,4,16,17]. Clinically, immediate lymphatic reconstruction is a preventative microsurgical lymphovenous anastomosis performed at the time of axillary lymph node dissection that is intended to mitigate lymphedema risk from 30% to 4–12% [2,3,6,7,14,18,19,20,21,22,23,24,25,26,27]. Translational experimental approaches to prevent lymphedema have included 9-cis retinoic acid, topical tacrolimus, and Prox1 [1,16,17,28].

“Ischemic preconditioning,” also known as the “vascular delay phenomenon,” is a clinically applied concept involving intentional, staged partial disruption of arteries for controlled ischemia to increase tissue viability for future ischemic insults. In reconstructive surgery, intentional partial vascular disruption of flaps followed by staged complete flap transfer 7–10 days later is common and referred to as the “vascular delay phenomenon” [29]. Cardiac applications of ischemic preconditioning have been exhibited with brief myocardial ischemia, with subsequent reperfusion conveying protective effects [29,30,31]. Similar ischemic preconditioning applications have been demonstrated in the liver and kidney [32,33].

Although vascular preconditioning with partial ischemia has shown protective effects across multiple organs, a similar corollary for lymphatic vessels remains unknown. The purpose of this study was to evaluate whether lymphatic preconditioning with staged disruption of lymphatics in the murine hindlimb lymphedema model can be protective against lymphedema.

## 2. Results

**Figure 1:**
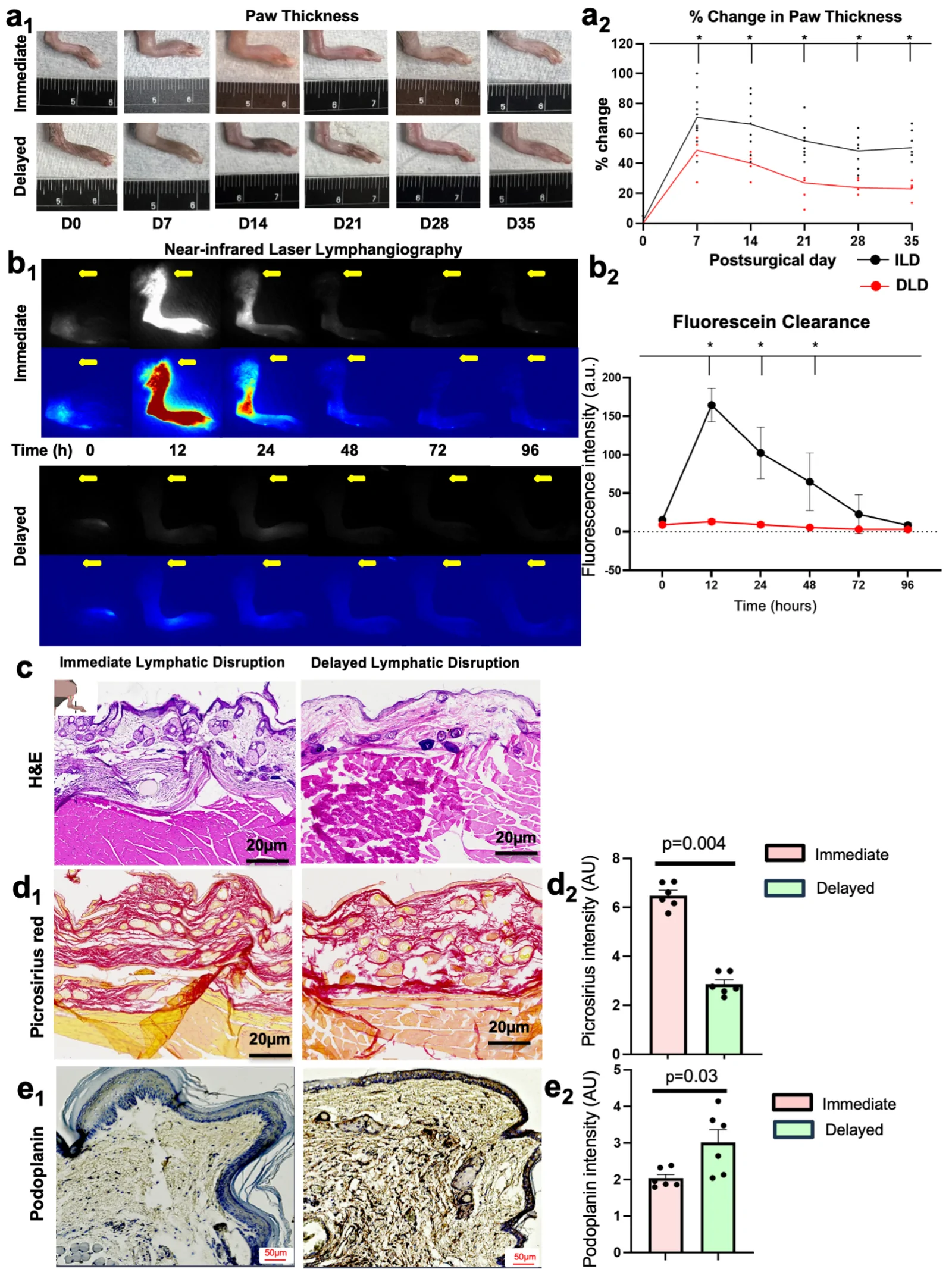
Lymphatic preconditioning in the murine hindlimb lymphedema model. The murine hindlimb model consists of preoperative irradiation, circumferential skin excision, lymphatic disruption and popliteal lymph node excision. Assessment of postsurgical lymphedema by (**a**) paw thickness measurement and (**b**) near-infrared lymphangiography, respectively. Representative images of paw thickness (**a1**) on the day of radiation and peak paw thickness swelling at Day 14 and Day 35 (n = 12). The percent change in paw thickness (**a2**) in the immediate lymphatic disruption (ILD) group (**black**) and delayed lymphatic disruption group (DLD) (**red**). Indocyanine green (2.5 mg/mL) was subcutaneously injected into the plantar aspect of the irradiated, lymphedematous hindlimb and non-operated hindlimb (**b1**). Post-injection time-lapse images were captured for 96 h (**b2**). Clearance in the immediate lymphatic disruption hindlimb group (**black**) compared to the delayed lymphatic disruption group (**red**) (Statistics: Mann–Whitney U test). Lymphatic transection level (**yellow arrow**). Representative histology images of murine paw tissue stained with (**c**) hematoxylin and eosin (H&E), (**d1, d2**) picrosirius red and (**e1, e2**) podoplanin, comparing the immediate lymphatic disrupted paw with the delayed lymphatic disrupted paw at Day 35 (Statistics: unpaired *t*-test). A *p*-value (*) < 0.05 was considered statistically significant.

The study included 12 mice (6 male, 6 female). Group 1 comprised 6 mice that underwent immediate lymphatic disruption, and Group 2 comprised 6 mice that underwent delayed lymphatic disruption (Figure 1). The average paw thickness of Group 1 on Day 14 post-surgery was 3.5 ± 0.3 cm compared to 2.9 ± 0.2 cm in Group 2 (*p* = 0.0003). Paw thickness on Day 35 was 3.2 ± 0.2 cm in Group 1 compared to 2.6 ± 0.1 cm in Group 2 (*p* = 0.0008). Near-infrared laser lymphangiography performed 24 h post-injection on Day 35 demonstrated a signal intensity of 102.4 ± 33.4 AU in Group 1 compared to 9.4 ± 3.3 AU in Group 2 (*p* = 0.0035). After subtraction of background fluorescence (≤0.22 AU per image), fluorescence intensity at 24 h remained lower in Group 2 (*p* = 0.0034) (Supplemental Figure S2). Immunohistochemical staining revealed an abundance of the lymphatic endothelial-specific marker podoplanin in the delayed lymphatic disruption group compared to the immediate lymphatic disruption group at Day 35 post-surgery (*p* = 0.03). When picrosirius red was used as a marker of collagen deposition at Day 35, less signal intensity was observed in the delayed lymphatic disruption group compared to the immediate lymphatic disruption group (*p* = 0.004).

## 3. Discussion

Secondary lymphedema is chronic swelling from lymphatic dysfunction [1,2,4,15,34,35,36,37,38,39,40,41,42,43,44,45]. Lymphedema typically occurs following lymph node dissection in patients who received radiotherapy for breast cancer or melanoma [1,2,4,5,15]. Lymphedema may occur in up to 30% of breast cancer patients who undergo axillary lymph node dissection [2,3,6,7,9,13,14,18,19,20,21,22,23,24,25,26,31,32,46,47,48,49,50,51,52,53,54,55,56,57,58]. Immediate lymphatic reconstruction is a preventative microsurgical technique to reduce lymphedema frequency [2,3,6,7,14,18,19,20,21,22,23,24,25,26,27].

The murine hindlimb is a preclinical animal model used to study lymphedema [2]. The murine hindlimb model involves preoperative irradiation of the hindlimb followed by subdermal lymphatic disruption and popliteal lymph node excision, resulting in consistent, sustained limb swelling up to 90 days post-surgery [2]. Although the rodent tail model of lymphedema is frequently used, the hindlimb model is more clinically translatable and has been shown to give consistent, reproducible swelling, particularly after irradiation [2,16].

Ischemic preconditioning or “vascular delay phenomenon” is a well-described concept to improve flap survival [29]. In the field of reconstructive plastic surgery, ischemic reperfusion has been frequently termed “vascular delay” [29]. Flaps are partially raised to allow for controlled ischemia [30,31,59,60]. Mechanisms of ischemic preconditioning for flap physiology include alteration in sympathetic tone, dilation of choke vessels, changes in tissue metabolism, and neovascularization [29]. After allowing for typically 7–10 days, the flap harvest is completed with resultant improved flap perfusion and survival [30,31,59,60]. Vascular delay is frequently used for random flaps and pedicled flaps such as transverse rectus abdominus muscle flap [61]. Cardiac applications of ischemic preconditioning are well-developed [30,31,60]. Subjecting myocardial cells to brief episodes of ischemia followed by reperfusion conveys protective effects against lethal myocyte death with sustained ischemic insult via slowing the rate of ATP depletion [30]. A randomized controlled trial found improved microvascular endothelial cell function mediated by enhanced prostacyclin production [31]. Hepatology studies showed enhanced hepatocellular proliferation with increased levels of Cyclin D1 with ischemic preconditioning [62]. Ischemic preconditioning activates angiogenic genes and promotes stem cell survival [63,64]. VEGF upregulation is induced by ischemic preconditioning in cholangiocytes in liver transplantation [33]. Renal ischemic preconditioning also has been shown to be protective against injury, reducing fibrosis and alpha-smooth muscle actin expression in kidneys 15 days after perfusion in animal models [32].

The concept conveying intentional, protective lymphangiogenic effects with staged, partial lymphatic disruption is a novel concept presented in this study. We propose that similar principles of ischemic preconditioning can be applied to lymphatic vessels for protection against lymphedema.

This study provides evidence for a novel concept of “lymphatic preconditioning,” with staged partial disruption of lymphatics being protective of lymphatic function. Animals exhibited less paw swelling in their hindlimbs when extensive lymphatic vessels and injury to lymph nodes were phased rather than performed at once. Near-infrared laser lymphangiography demonstrated preservation of lymphatic function with staged disruption. Lymphatic preconditioning led to greater lymphatic abundance with increased podoplanin staining. Picrosirius red staining was consistent with less scarring/fibrosis following lymphatic preconditioning. This suggests that the mechanism may involve promoting lymphangiogenesis in a manner similar to ischemic preconditioning, activating angiogenic genes and promoting stem cell survival [30,31,60,64]. Reduced fibrosis may indicate less inflammatory effects resulting from staged lymphatic disruption.

In line with established approaches, in lymphedema animal models, combination approaches (irradiation, skin incision and lymph node dissection) are generally preferred to achieve consistent, sustained lymphedema in mouse hindlimb models [11,16,60,61,63,65,66,67,68,69,70,71,72,73,74,75,76,77,78,79]. This hindlimb lymphedema model more closely parallels clinical lymphedema, which involves incision, lymphadenectomy, lymphatic disruption, and typically radiation. In our previous publication, we optimized this murine hindlimb lymphedema model and compared the hindlimb lymphedema model with and without radiation [2]. We found that radiation of the murine hindlimb model results in sustained lymphedema compared to non-irradiated mice and is therefore a more effective model. Our experimental sequence was selected based on the prior literature and our prior studies demonstrating that radiation followed by surgery one week later reliably induces sustained lymphedema in this model. Importantly, radiation alone, without subsequent surgery, does not induce lymphedema (Supplemental Figure S1).

Previous studies have demonstrated that tissue hypoxia can contribute to lymphangiogenesis through hypoxia-responsive signaling pathways [80,81,82]. In experimental models of lymphatic regeneration, hypoxia-induced stabilization of HIF-1α has been shown to regulate lymphangiogenic cytokine expression, including VEGF-C, and inhibition of HIF-1α delays lymphatic repair [82]. In addition, HIF-1α-dependent regulation of VEGF-C and VEGFR-3 signaling has been implicated in lymphangiogenesis in several experimental and pathological settings [80,81,83]. Collectively, these findings suggest that, similar to angiogenesis, lymphatic remodeling and regeneration may be influenced by the hypoxic microenvironment [80,81,82]. However, because hypoxia and HIF-related signaling were not directly evaluated in the present study, the extent to which these mechanisms contribute to the lymphatic response observed in our model remains to be determined.

Importantly, our study was designed to characterize lymphatic regeneration following lymphatic preconditioning and was not specifically designed to establish a causal relationship between hypoxia and lymphangiogenesis. Future studies incorporating tissue- and cellular-level assessment of hypoxia, together with evaluation of lymphangiogenic signaling, will be important for determining whether hypoxia directly contributes to lymphatic regeneration following preconditioning and for further defining the underlying biological mechanism. A limitation of our study includes interpretation of ICG fluorescence signals. ICG lymphography predominantly assesses superficial lymphatic transport and may not accurately represent the regeneration and functional recovery of deeper lymphatic vessels. Therefore, the interpretation of ICG fluorescence signals as evidence of lymphatic regeneration should be made with caution.

This study introduces the novel concept of lymphatic preconditioning for preservation of lymphatic function. This proof-of-concept study opens a frontier for mechanistic studies and potential translational clinical applications for protective effects to prevent lymphedema. Oncological treatment of cancer (e.g., breast cancer and melanoma) that may require lymph node excision may benefit from partial lymph node removal, marking the remaining lymph nodes, and staged operations to remove the remainder of the lymph node basin. A potentially powerful translational application could be staging lymphadenectomy by maintaining select lymph nodes during resection, marking them with a clip, and returning to the operating room a week later to complete the lymphadenectomy. This would allow for a complete resection while facilitating a “lymphatic delay phenomenon.”

## 4. Materials and Methods

Experiments were conducted following approval by the Indiana University Institutional Animal Care and Use Committee (IACUC). Male and female C57BL/6 mice that were 9–10 weeks of age (21–23 g) at the time of treatment were used. Mice were purchased from Jackson Laboratory (Bar Harbor, ME, USA).

**Figure 2:**
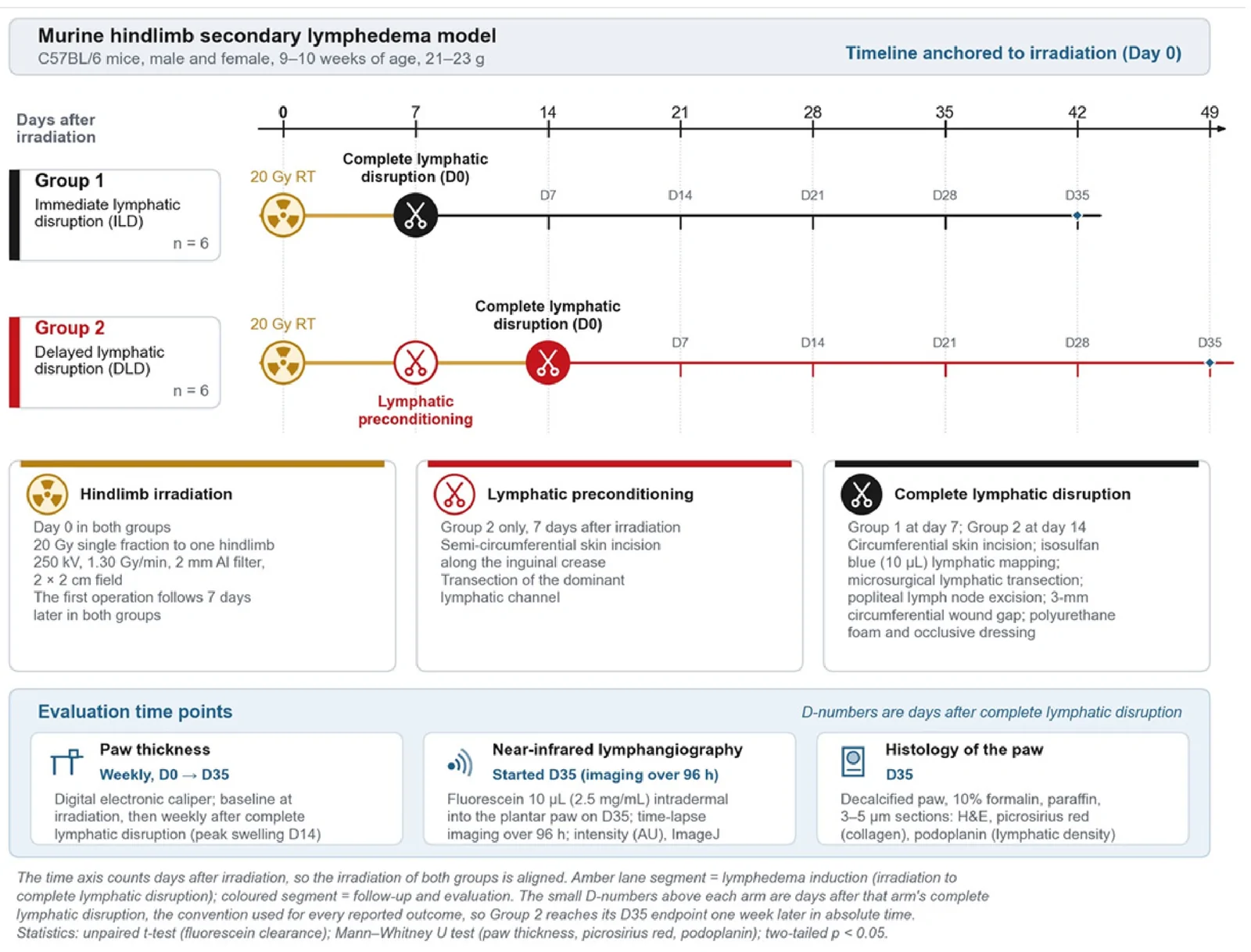
Schematic diagram. Timeline of secondary lymphedema induction, treatment protocol, and evaluation time points.

The murine hindlimb lymphedema model was utilized (Figure 2) [2]. Once sedated with isofluorane, mice were positioned on plexiglass within a flat-beam field, and one hindlimb of each mouse was irradiated with 20 Gy of 250 kVp X-rays (2 × 2 cm field size) using a Precision X-ray machine (Precision X-Ray Inc., North Branford, CT, USA) with a 2 mm Al filter (dose rate = 1.44 Gy/min) [2]. Dosimetry was performed using a PTW Model N30013 Farmer-type ionization chamber (Freiburg, Germany) in conjunction with a Max 4000 Plus electrometer (Standard Image, Middleton, WI, USA). Mice were monitored during irradiation to ensure their feet did not move out of the radiation field; breathing was also monitored so anesthesia could be adjusted as necessary to keep the mouse sedated.

Group 1 (immediate lymphatic disruption) underwent a circumferential skin incision, complete lymphatic disruption, and popliteal lymph node excision in a single-stage operation one week post-radiation (Figure 2). Group 2 (delayed lymphatic disruption) underwent a two-stage operation. In Group 1 (control), an incision was made at the proximal hindlimb with reflected skin flaps to expose the underlying subcutaneous soft tissue one week post-radiation. Intradermal injection of isosulfan blue dye (10 µL) into the mouse paw was performed to identify lymphatics. Lymphatic vessels were cut using microsurgical scissors. Popliteal lymph nodes were excised. Skin re-approximation created a 3 mm circumferential wound gap for subdermal lymphatic discontinuity. A 3 mm polyurethane foam was circumferentially applied to the proximal hindlimb wound. An occlusive dressing was applied over the surgical wound. Wound management using polyurethane foam was standardized between Group 1 and Group 2.

In Group 2 (delayed lymphatic disruption), the first operation one week post-radiation included a semi-circumferential skin incision along the inguinal crease and transection of the dominant lymphatic channel. The second operation occurred 7 days later and included a circumferential skin incision, lymphatic transection, and popliteal lymph node excision. Paw thickness was measured at weekly intervals using a digital electronic caliper following complete lymphatic disruption. The 35-day endpoint was selected based on our previous studies and established characteristics of this experimental model [2,4]. At this time point, the postoperative inflammatory response has largely subsided, while sustained lymphatic dysfunction and associated tissue remodeling are established, allowing assessment of relatively stable lymphatic changes rather than the immediate effects of surgery.

Near-infrared laser lymphangiography was used for functional lymphatic assessment on Day 35 following complete lymphatic disruption [1,2,4]. Indocyanine green dye (10 µL, 2.5 mg/mL) was intradermally injected into the plantar paw. Fluorescence imaging was captured over 96 h using near-infrared laser lymphangiography (SPY Fluorescence Imaging, Stryker, Kalamazoo, MI, USA). ImageJ (version 1.54g, Bethesda, MD, USA) was used to calculate fluorescence signal intensities in arbitrary units (AU). Background fluorescence was measured in a fixed region outside the hindlimb in each image and subtracted. The spatial distribution of clearance was assessed by automated image analysis in Python (version 3.12) using PyTorch (version 2.14) on a graphics processing unit (GeForce RTX 5090, NVIDIA Corporation, Santa Clara, CA, USA). Images of each hindlimb were aligned to the 12 h image, and an exponential clearance model was fitted at every pixel to generate maps of local clearance half-life. No blank or missing data points were present for the mice included in the lymphangiography analysis. Indocyanine green clearance was determined by plotting the intensity of fluorescence over time.

Histological examination included decalcified murine paw tissues (10% formalin fixation, paraffin-embedded). Histology samples were sectioned in 3–5 μm intervals and stained with hematoxylin and eosin (H&E), podoplanin for lymphatic density, and picrosirius red (collagen deposition/scarring) at Day 35 following complete lymphatic disruption.

Statistical analyses were performed using GraphPad Prism (GraphPad Software, version 10.6.1, Boston, MA, USA). Parametric continuous variables were compared using unpaired samples *t*-tests for indocyanine green dye clearance. Non-parametric continuous variables were calculated using Mann–Whitney U tests for comparison of paw thickness and picrosirius red and podoplanin intensities between groups. Two-tailed values of *p* < 0.05 were considered statistically significant.

## Data Availability

The original contributions presented in this study are included in the article/Supplementary Material. Further inquiries can be directed to the corresponding author.

## Supplementary Materials

The following supporting information can be downloaded at https://www.mdpi.com/article/10.3390/ijms27209125/s1.
