# Supplementary material for: Lymphatic Preconditioning for Preservation of Lymphatic Function
Source: Int J Mol Sci. 2026 Oct 15;27(20):9125. doi: 10.3390/ijms27209125 (PMC13704935; doi:10.3390/ijms27209125)
Supplement: Supplementary file 1 [file ijms-27-09125-s001.zip › ijms-4540883-supplementary.pdf]

Supplemental Figure S1

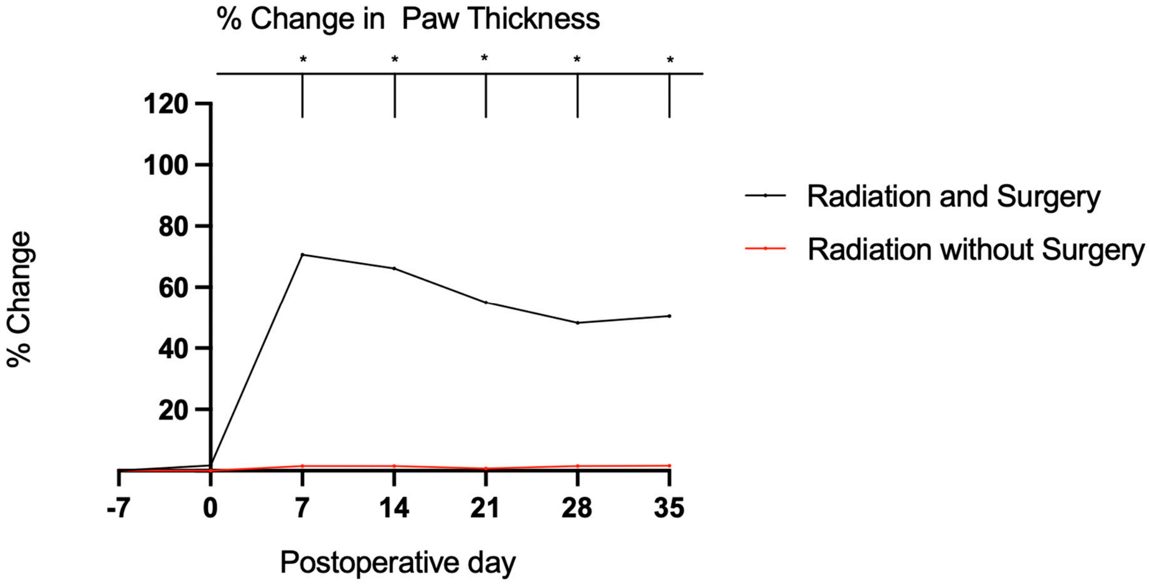

Supplemental Figure S1. The percent change in paw thickness in the radiation with hindlimb surgery group (**black**) and the radiation without hindlimb surgery group (DLD) (**red**). Radiation followed by hindlimb surgery one week later reliably induces sustained lymphedema in this model. Importantly, radiation alone, without subsequent hindlimb surgery, does not induce lymphedema. A *p*-value (*) <0.05 was considered statistically significant using the Mann–Whitney U Test.

Supplemental Figure S2

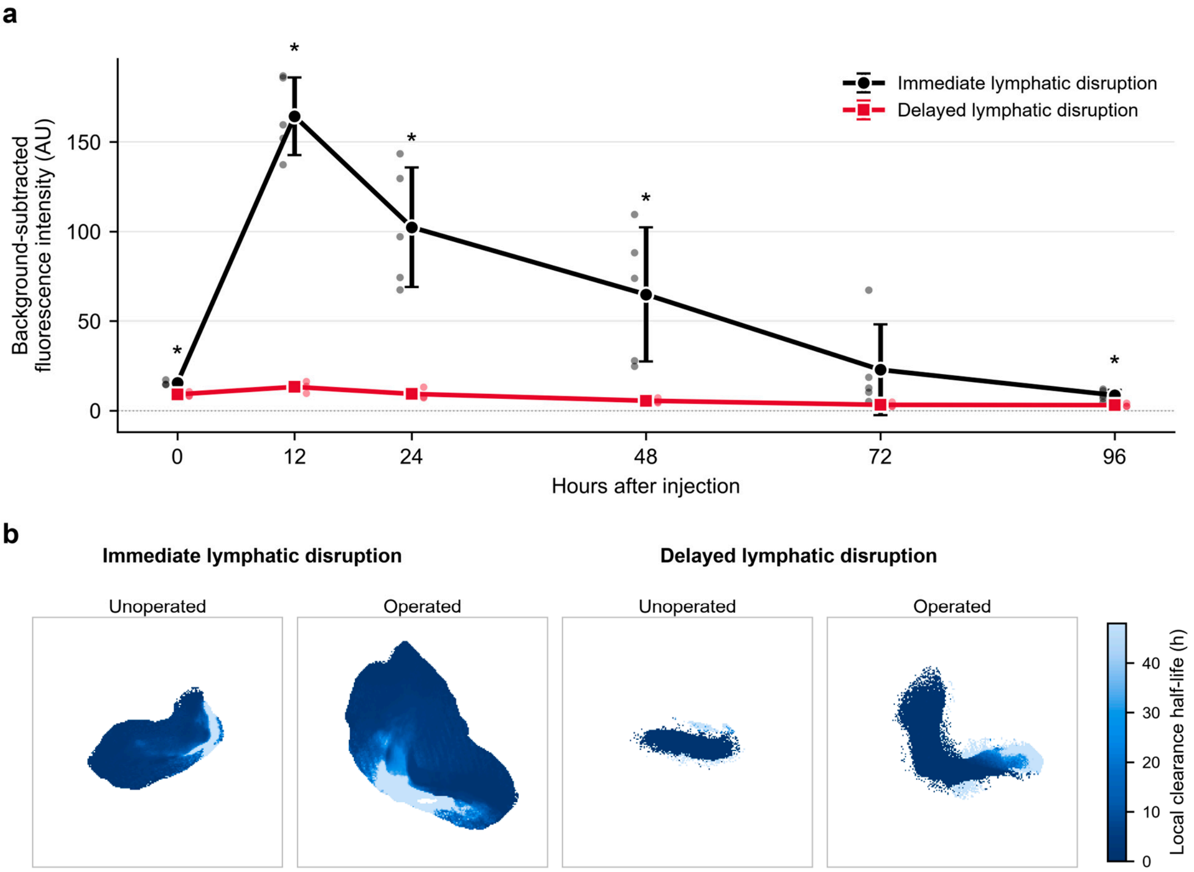

Supplemental Figure S2. Background-subtracted near-infrared laser lymphangiography and spatial analysis of clearance. (a) Fluorescence intensity of the operated hindlimb after subtraction of background fluorescence in the immediate lymphatic disruption (ILD) and delayed lymphatic disruption (DLD) groups from 0 to 96 h after intradermal injection of indocyanine green on Day 35. Background fluorescence was measured in a fixed region outside the hindlimb in every image (≤0.22 AU) and subtracted. Points represent individual measurements; lines represent mean ± standard deviation (ILD, n=5 [n=3 at 0 h]; DLD, n=3). At 24 h, fluorescence intensity was 102.4 ± 33.4 AU in the ILD group compared to 9.3 ± 3.3 AU in the DLD group (p=0.0034). (b) Local clearance half-life at each pixel of the unoperated and operated hindlimbs of a representative mouse from each group, calculated by automated image analysis. Images

from 12 to 96 h were aligned to the 12 h image, the background was subtracted, and an exponential clearance model was fitted at every pixel. Darker pixels cleared faster. All maps share one color scale. ILD, black; DLD, red. *$p < 0.05$ (unpaired t-test). AU, arbitrary units.
